# Neuroprotective effects of *Opuntia ficus-indica* fruit in high-fat-diet-induced obese mice: evidence for a role of the gut microbiota

**DOI:** 10.3389/fnut.2026.1909657

**Published:** 2026-08-05

**Authors:** Pasquale Calvi, Simona Terzo, Marta Giardina, Salvatore Timeo, Laura Palumbo, Nicolò Ricciardi, Giuditta Gambino, Pierangelo Sardo, Alessandro Massaro, Domenico Nuzzo, Pasquale Picone, Flavia Mulè, Antonella Amato

**Affiliations:** 1 Department of Biological, Chemical and Pharmaceutical Sciences and Technologies (STEBICEF), University of Palermo, Palermo, Italy; 2 Institute for Biomedical Research and Innovation (IRIB), Palermo, Italy; 3 Biomedicine, Neuroscience and Advanced Diagnostics (Bi.N.D.), University of Palermo, Palermo, Italy

**Keywords:** gut microbiota, high-fat diet, neuroprotection, obesity, *Opuntia ficus-indica*

## Abstract

Obesity and high-fat diet (HFD) consumption are associated with cognitive decline and increased risk of neurodegenerative disorders, including Alzheimer’s disease (AD). Gut microbiota dysbiosis is a key factor for obesity-related brain dysfunctions. *Opuntia ficus-indica* fruit (OFIF) has shown beneficial metabolic and neuroprotective effects, but the contribution of the gut microbiota has not been explored yet. This study investigated whether OFIF exerts neuroprotective effects in HFD mice through microbiota-dependent mechanisms. C57BL/6 mice were fed a standard diet or HFD for 16 weeks, with or without OFIF supplementation. A subgroup of HFD + OFIF mice received a broad-spectrum antibiotic cocktail to deplete gut microbiota. Metabolic parameters, cognitive performance, brain morphology, AD–related gene expression (PCR array), and gut microbiota composition were assessed. OFIF supplementation improved insulin resistance in HFD-mice, also in the presence of antibiotics, suggesting microbiota-independent metabolic benefits. In contrast, OFIF prevented HFD-induced reduced brain weight, neuronal damage, and partially restored cognitive impairment, effects that were abolished following microbiota depletion. At the molecular level, OFIF modulated the expression of multiple AD-related genes in the cortex and hippocampus, including pathways involved in amyloid processing, synaptic function, and lipid metabolism. These transcriptional changes were absent in antibiotic-treated mice. Microbiota analysis revealed that OFIF positively modulated gut microbiota composition, leading to enrichment of genera such as *Duncaniella* and *Paramuribaculu*m, which positively correlated with brain structural. In conclusion, our findings suggest that microbiota is a critical mediator of OFIF-driven brain protection and supports the potential of OFIF as a dietary strategy to counteract obesity-associated cognitive decline.

## 1 Introduction

Accumulating evidence suggests that aging individuals with obesity develop cognitive impairment and susceptibility to Alzheimer’s disease (AD) (1). Indeed, the consumption of a high fat diet (HFD) is involved not only in obesity development and its related dysfunctions, but also it impacts the brain health (2). Nowadays, intake of saturated fat rich diet is widely considered as a driving factor for cognitive impairment and AD development (3). AD is a neurodegenerative disorder characterized by progressive cognitive decline and memory impairment (4). The neuropathological hallmarks of AD include amyloid-β (Aβ) accumulation resulting from amyloid precursor protein (APP) cleavage via β-site APP-cleaving enzyme 1 (BACE1) and *γ*-secretase, synaptic loss, and the deposition of hyperphosphorylated tau protein forming neurofibrillary tangles in the cerebral cortex and hippocampus (5). Aging, genetic and environmental factors contribute to alterations that shift APP cleavage toward the amyloidogenic pathway rather than the normal non-amyloidogenic secretory process (5). In addition, a variety of factors including acetylcholine deficiency, glutamate imbalance, oxidative stress, neuroinflammation, insulin resistance, mitochondrial dysfunction, cholesterol homeostasis, gut dysbiosis are implicated in AD pathogenesis (6).

Increasing evidence emphasizes that cognitive decline should be considered a major consequence of living in an obesogenic environment (7). In healthy rodents, HFD disrupts performance in learning and memory tasks and, in some cases, induces AD-like pathological changes (8–12). Therefore, HFD-fed mice for a long period represent a good experimental model to understand the link between obesity and neuronal damage leading to neurodegeneration/AD and to explore the potential of neuroprotective substances. Insulin resistance, oxidative stress, neuroinflammation, mitochondrial dysfunction, gut dysbiosis, all key consequences of obesity, are thought to interact and contribute to the neurodegenerative conditions and cognitive decline seen in AD (13). Moreover, increasing evidence highlights the gut–brain axis as a central mechanism linking obesity to neurological outcomes (14). Indeed, HFD-induced obesity disrupts gut microbial homeostasis, leading to decreased microbial diversity, enrichment of pro-inflammatory species, and reduced synthesis of health-promoting metabolites. Such alterations in gut microbiota composition are now considered major contributors to AD-like cognitive deficits (14). Accordingly, targeting the intestinal microbiota in obese individuals may offer a promising therapeutic strategy for the management of obesity and its associated metabolic disorders (15).

Recently, we demonstrated that 4-week supplementation with lyophilized yellow prickly pear fruit (*Opuntia ficus-indica* fruit; OFIF) improves glucose metabolism and it counteracts insulin resistance in HFD mice (16). Moreover, administration of OFIF at a dose of 14 mg/kg for 1 month in HFD-fed rats attenuates obesity-associated cognitive impairments and metabolic dysfunctions (17). However, whether long-term dietary supplementation with the whole fruit provides sustained neuroprotection throughout chronic obesogenic feeding remains unknown. Moreover, the contribution of the gut microbiota to the effects of OFIF on brain homeostasis has not yet been investigated.

Therefore, the aim of the present study was to determine whether long-term OFIF alleviates HFD-induced gut dysbiosis, cognitive performance, and neurodegeneration/AD associated pathological features. To this end, mice were aged and fed an HFD supplemented with OFIF for 16 weeks, and a subset of animals were treated with a broad-spectrum antibiotic cocktail to abrogate the gut microbiota and thereby assess microbiota role in mediating the effects of OFIF.

## 2 Materials and methods

### 2.1 Animals and diet

Male mice of the C57BL/6 strain, purchased at 4 weeks of age from Envigo Laboratories (S. Pietro al Natisone, Udine, Italy), were used in the present study. The animals (*n* = 32) were housed in the Advanced Technologies Network (ATeN) animal facility under controlled environmental conditions, including 12 h light/12 h dark cycle, constant temperature (22 ± 2 °C) and relative humidity (55% ± 5%) with ad libitum access to food and water. All animal housing procedures and experimental protocols were conducted in accordance with Italian Legislative Decree No. 26/2014 and the European Directive 2010/63/EU for animal experimentation and were approved by the Italian Ministry of Health (Authorization No. 395/2024-PR, issued on May 6, 2024).

The preparation of lyophilized *Opuntia ficus-indica* fruit powder (OFIF) was obtained from the yellow cultivar of *Opuntia ficus-indica* grown in San Cono (Sicily). The fruits were peeled, finely chopped, and weighed. Subsequently, 100 g of pulp were homogenized and lyophilized in the presence of 2.5% food-grade β-cyclodextrin.

After 1 week of acclimatization, mice (two animals per cage) were assigned to four experimental groups (*n* = 8 per group), fed for 16 weeks as follows: the STD group received a standard diet (20% protein, 70% carbohydrates, 10% fat; ref. 4RF25, Mucedola, Milan, Italy); the HFD group received a high-fat diet (20% protein, 20% carbohydrates, 60% fat; PF4215, Mucedola, Milan, Italy); the HFD + OFIF group received the HFD supplemented with lyophilized *Opuntia ficus-indica* fruit (12 g/kg diet); and the HFD + ATB + OFIF group received the HFD supplemented with OFIF and an antibiotic cocktail administered in drinking water (ampicillin 1 g/L, neomycin 1 g/L, vancomycin 0.25 g/L, and metronidazole 1 g/L). Antibiotic treatment was maintained throughout the experimental period to deplete the gut microbiota. The OFIF-supplemented diet was custom-prepared by Mucedola and was isocaloric with the HFD. The dose of OFIF (12 g/kg diet) was chosen based on a nutritionally relevant human intake of the fruit (four fruits/day, corresponding to 75 g of lyophilized product) and normalized to the average daily food intake of mice. Body weight and food intake were recorded weekly throughout the 16-week experimental period. At the end of the study duration, blood glucose concentration was measured from a drop of blood collected from the tail vein using a glucometer (Gluco-Men LX meter, Menarini, Florence, Italy) in overnight fasting mice. Subsequently, animals were weighed and sacrificed. Intracardiac blood samples were collected, and brains were excised and weighed. Blood was centrifuged at 3,000 rpm for 15 min at 4 °C to obtain plasma that was stored at −80 °C. Brain samples were then processed as follows: a cerebral portion was coronally sectioned, fixed in formalin, and used for histological analyses; the remaining tissue was dissected to separate cortex and hippocampus, stored in sterile Eppendorf tubes, and frozen at −80 °C for subsequent biomolecular analyses. Moreover, 6 h prior to sacrifice, mice were individually housed in sterile cages, and approximately 300 mg of fresh fecal samples were collected from each animal. Samples were immediately stored at −80 °C for subsequent analyses.

### 2.2 Determination of plasma insulin

Plasma insulin concentration was determined using a commercially available Enzyme-Linked Immunosorbent Assay (ELISA) kit (Mercodia, Sylveniusgatan 8A, Uppsala, Sweden), according to the manufacturer’s instructions. Insulin resistance was assessed by calculating the HOMA index (HOMA-IR) using the following formula: HOMA-IR = [fasting insulin concentration (mU/L) × fasting glucose concentration (mg/dL) × 0.05551]/22.5.

### 2.3 Histological analysis of the cortex

Cortical tissue samples were fixed in 4% formaldehyde in phosphate-buffered saline (PBS), dehydrated in graded ethanol solutions (70, 80, 96%), cleared in xylene and embedded in paraffin blocks, that were allowed to solidify at room temperature. Sections of 5 μm thickness were obtained using a rotary microtome (YIDI YD315, Jinhua, Zhejiang, China) and mounted on glass slides. Hematoxylin and eosin (H&E) staining was performed on fixed, paraffin-embedded mouse brain sections according to standard histological procedures to obtain a morphological evaluation of brain tissue architecture. Stained sections were examined using a light microscope (Leica DMLB, Meyer Instruments, Houston, TX, United States) equipped with a digital camera (DS-Fi1, Nikon, Florence, Italy). Viable and dead neurons were quantified in three images per sample captured at 200 × magnification. Neurons were classified as viable based on preserved morphology, defined by the presence of a clearly visible nucleus and nucleolus, along with lightly stained cytoplasm. Dead neurons were identified by intense cresyl violet staining, absence of a distinct nucleolus, and the presence of pericellular vacuolation. Cell counts were performed in a blinded manner by two independent investigators using NIS-Elements Basic Research (Nikon, Florence, Italy). The average of the counts was calculated and expressed as the percentage of viable cells per area according to the following formula: % viable cells = [viable cells/(viable cells + dead cells)].

### 2.4 RT^2^ profiler PCR array

Gene expression changes in cortex and hippocampus of mice belonging to four animal groups were assessed using the Mouse Alzheimer’s Disease RT^2^ Profiler PCR Array (PAMM-057Z, Qiagen, Monza, Italy) in a 96-wellplate format. Each array probes 84 pathway-related genes, 5 housekeeping reference genes (*Actb, B2m, Gapdh, Gusb, Hsp90ab1*), 1 mouse genomic DNA contamination control, 3 reverse transcription controls (to check reverse transcription efficiency) and 3 PCR positive controls (to assess array reproducibility). Tissues were homogenized with a hand-held homogenizer, and total RNA was extracted using the PureLink™ RNA Mini Ki (Invitrogen™, Thermo Fisher Scientific, Waltham, MA, United States). To synthesize cDNA, 0.5 μg RNA was reversed transcribed using the RT^2^ First Strand Kit (Qiagen, Monza, Italy #330404). PCR reactions were performed according to manufacturer’s instructions and run on a QuantStudio™ 3 Real-Time PCR System (Applied Biosystems™, Thermo Fisher Scientific, Waltham, MA, United States). Analysis of gene expression data was performed using Qiagen’s online platform (RT^2^ Profiler PCR Arrays & Assays Data Analysis software).^1^ All genes were normalized to a minimum of three reference genes. Data showing at least a two-fold change with a *p* ≤ 0.05 were considered significant. Validation of the RT^2^ PCR Array results was performed by real-time PCR with predesigned primers for *Abca1*, *Apbb2*, *Apbb1*, and *Bche* purchased from Qiagen (Monza, Italy, assay IDs: QT00165690, QT00111125, QT00125433, QT01057154).

### 2.5 Behavioral assessment

The Open Field (OFT) and Object Recognition (ORT) tests were evaluated using behavioral tracking software (AnyMaze, Version 7.20) and analyzed by a trained scientist who was unaware of the experimental grouping. The OFT was employed to assess locomotor and exploratory behavior of the animals following established protocols (17–19). Mice were introduced into a square open field maze (44.5 × 44.5 cm), positioned facing the wall from a distance of 10 cm and were subsequently recorded for a duration of 10 min. Recorded activity parameters included Total distance covered, Immobility time and Number of line crossings. The Object Recognition test (ORT) is a reliable and widely employed method for evaluating learning and memory by assessing the declarative memory system in experimental models (17, 18). Initially, rodents were habituated to the arena (open field maze with the same characteristics as in OFT). On the test day, animals were presented with two identical objects placed at a fixed distance from each other. Following a 1-h retention period, one of the two objects was replaced with a novel one, while the other remained unchanged (“familiar object”). The objects were selected and 3D printed based on specific literature pertaining to this paradigm (20). The amount of time the animals spent exploring each object was tracked. In assessing the ORT, we considered the Total exploration time, the Exploration index (calculated as Latency (s) to the first investigation of the New Object divided by the total number of investigations of the New object + number of investigations of the Known object) and the Recognition index (RI%, representing the time spent exploring the novel object relative to the total time spent investigating both the familiar and novel one). Moreover, after a further 24-h retention period, one of the two objects (the familiar or the new one) presented during the 1-h retention test was replaced with a completely novel one, exploring the ability to retain information 24 h after the object presentation.

### 2.6 Gut microbiota composition

For gut microbiota analysis, faucal samples were collected from mice and stored at −80 °C until processing. Genomic DNA was extracted using the DNeasy^®^ 96 PowerSoil Pro Kit (Qiagen, Hilden, Germany) on the QIAcube HT automated platform, following the manufacturer’s protocols. The V3-V4 region of the 16S rRNA gene was amplified via PCR with modified primers described by Takahashi et al. (21). Amplicons were purified using Thermolabile Exonuclease I (New England Biolabs, Ipswich, MA, United States), diluted (1:2), and indexed with Nextera XT Index Kit (Illumina, San Diego, CA, United States). Libraries were normalized using the SequalPrep Normalization Kit (Thermo Fisher Scientific, Waltham, MA, United States), pooled, and further purified with Agencourt AMPure XP magnetic beads (Beckman Coulter, Brea, CA, United States). Sequencing was performed on the MiSeq platform (Illumina, San Diego, CA, United States) using V3 chemistry (2 × 300 bp paired-end reads). Sequencing data were processed using QIIME 2 (version 2023.7). Primer sequences were removed using Cutadapt (v.2023.7), and reads were denoised with DADA2 to generate amplicon sequence variants (ASVs). Taxonomic classification was performed using the SILVA v138 and GreenGenes2 v2022.10 reference databases.

### 2.7 Statistical analysis

The results are reported as mean ± Standard Error of the Mean (SEM). Statistical analyses were conducted by ANOVA, followed by Bonferroni’s multiple comparison *post-hoc* test using Prism 6.0, GraphPad (San Diego, CA, United States). Associations between fecal bacterial profiles and brain atrophy indices, hippocampal and cortical gene expression were assessed using Spearman’s rank correlation. Spearman’s rank correlation *p-*values were adjusted for multiple comparisons using the Benjamini–Hochberg false discovery rate correction. A *p* < 0.05 was considered indicative of statistical significance.

## 3 Results

### 3.1 Effects of OFIF on body weight, food intake and glucose homeostasis

**Figure 1:**
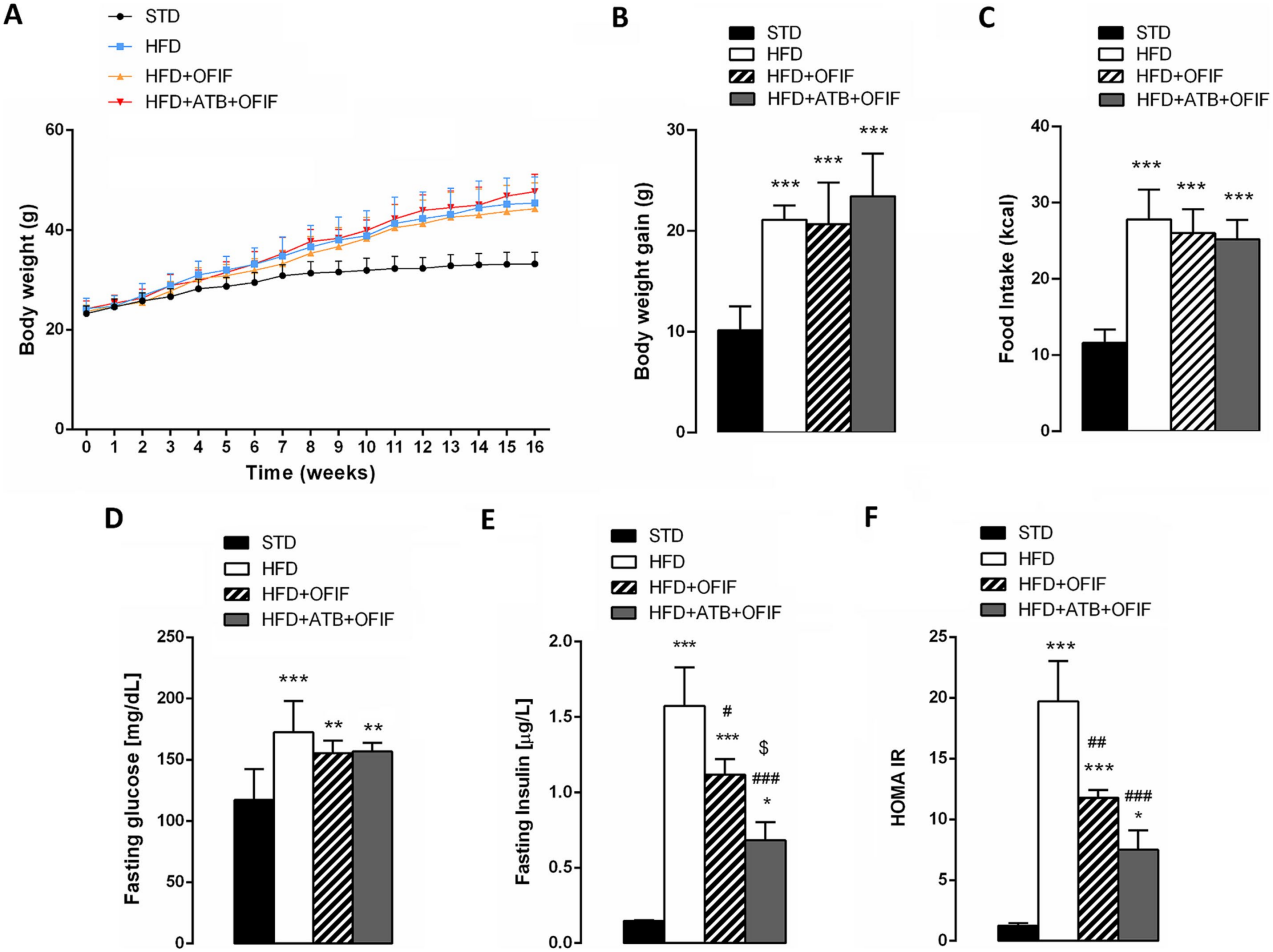
The impact of OFIF on body weight, caloric intake, and glucose homeostasis in the different groups of mice. The graphs present **(A)** the body weight of the animals during the experimental period; **(B)** the final body weight gain; **(C)** the average caloric intake per mouse; **(D)** plasma fasting glucose concentration (mg/dL); **(E)** plasma fasting insulin levels (μg/L); **(F)** HOMA-IR. Data are presented as mean ± SEM (*n* = 8). *p < 0.05, ***p < 0.001 versus the STD group; #*p* < 0.05, ##*p* < 0.01, ###*p* < 0.001 versus the HFD group; $*p* < 0.05 versus the HFD + OFIF group.

At the beginning of the feeding period, no significant differences in mean body weight were observed among the four experimental groups (STD, HFD, HFD + OFIF, and HFD + ATB + OFIF; Figure 1A). After 16 weeks of dietary intervention, body weight gain was significantly higher in all groups fed HFD compared with the STD group (Figure 1B). Caloric intake differed significantly between the STD and HFD groups; however, mice feeding HFD supplemented with OFIF or OFIF plus antibiotics assumed a similar amount of kilocalories compared with mice fed the HFD alone (Figure 1C).

Moreover, the HFD group exhibited significantly higher fasting glycaemia, insulin concentration, and Insulin resistance index (HOMA-IR) compared with the STD group (Figures 1D–F). In contrast, insulin levels and the HOMA-IR were significantly lower in both the HFD + OFIF and HFD + ATB + OFIF groups compared with the HFD group (Figures 1D–F).

### 3.2 Effects of OFIF on brain weight

**Figure 2:**
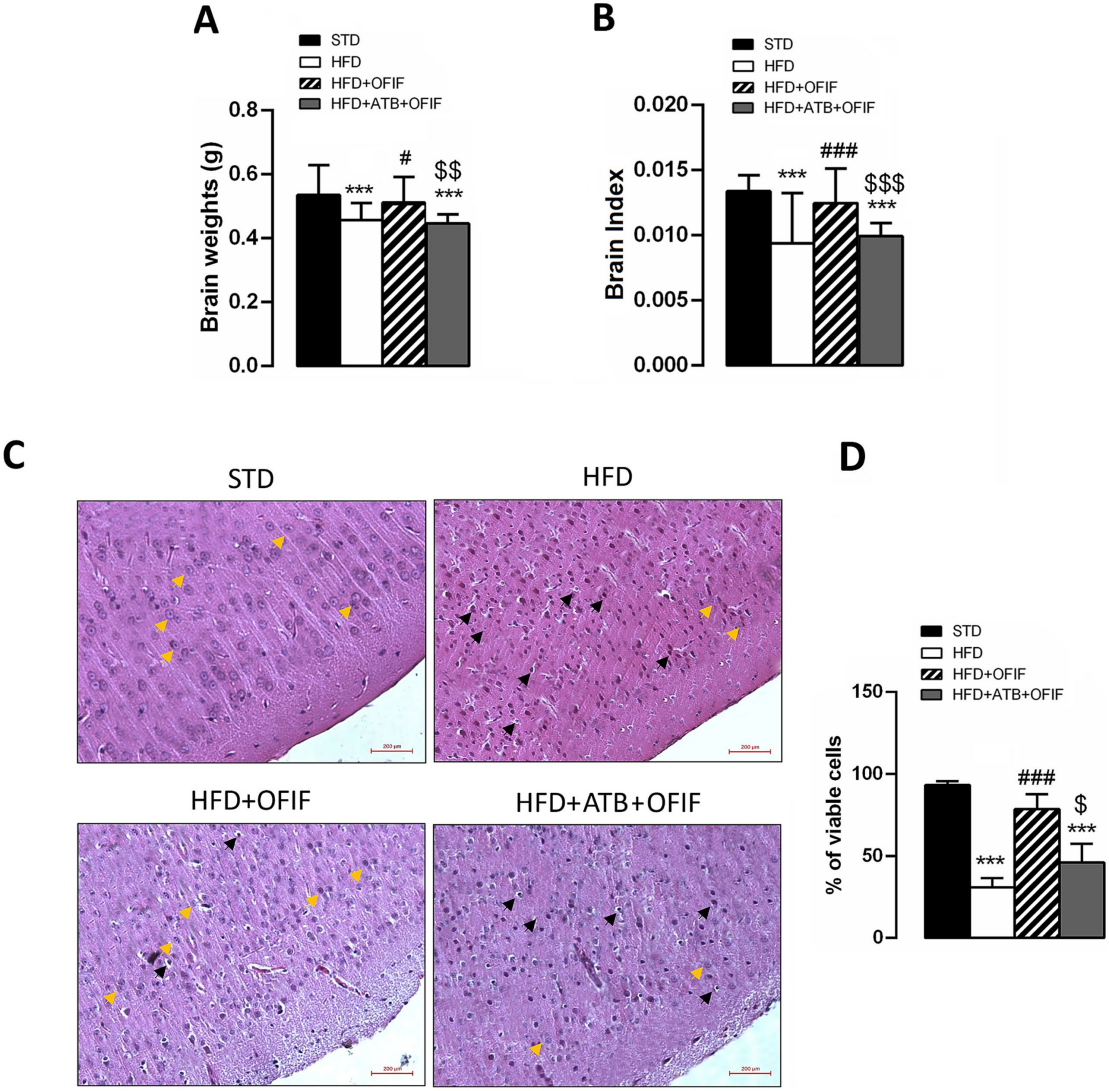
Impact of OFIF on cerebral cortex morphology in the different groups of mice. **(A)** Brain weight and **(B)** brain index (brain weight/body weight ratio), **(C)** cortical morphology (yellow arrows indicate viable cells and black arrows indicate dead cells), **(D)** histograms of the percentages of intact neurons in cortex areas in the different groups of mice (*n* = 8). Data are presented as mean ± SEM from each group. *p < 0.05 and ***p < 0.001 versus the STD group; #*p* < 0.05 and ##*p* < 0.01 versus the HFD group; ###*p* < 0.001 versus the HFD group; $*p* < 0.05 versus the HFD + OFIF group. Haematoxylin and eosin (H&E) staining was used to visualize the morphological changes of nerve cells in the cerebral cortex (20x).

Brain weights and the brain-to-body weight ratio were significantly lower in HFD mice compared to the STD group (Figures 2A,B). In contrast, both parameters in HFD + OFIF mice were comparable to those of STD mice. No improvement in reduction of brain weight was observed in antibiotics-treated mice. Histological examination of cortical sections revealed well-preserved brain architecture in the STD group, with high neuronal density (Figure 2C). In contrast, the HFD group exhibited clear hallmarks of neurodegenerative damage, including a reduced number of neurons, neuron disorganization, presence of vacuolization, some pyknotic nuclei and hypereosinophilic cytoplasm. Interestingly, mice receiving OFIF displayed a significant reduction in HFD-induced damage. The neuronal density and organization were better preserved than in the HFD group and more closely resembled those of the STD mice, suggesting a neuroprotective effect of OFIF. Conversely, group treated with antibiotics to deplete gut microbiota showed no evident improvement in the HFD-associated alterations, showing neurons with hypereosinophilic cytoplasm and pyknotic nuclei, consistent with ongoing neurodegeneration.

### 3.3 Effects of OFIF on cognitive performances

**Figure 3:**
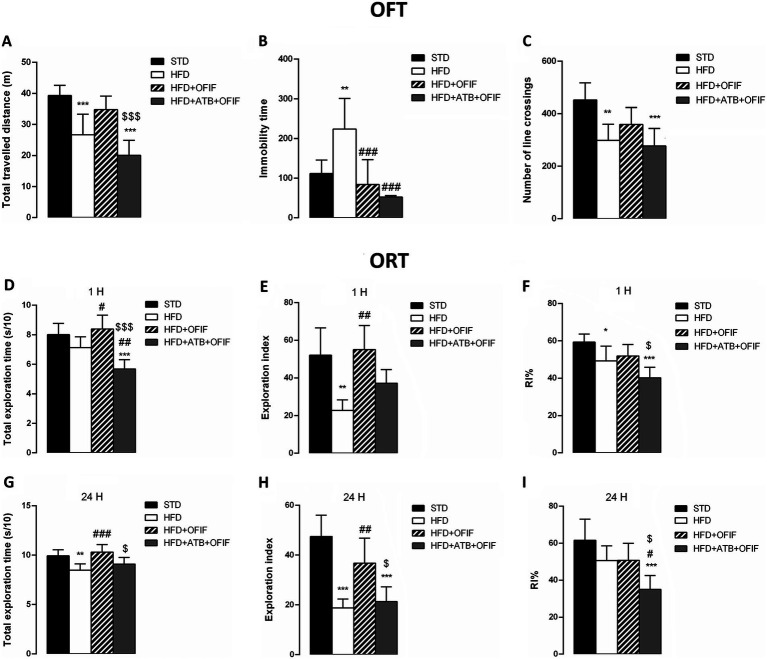
Impact of OFIF on cognitive performance in the different groups of mice. Open field test showing **(A)** total distance travelled; **(B)** immobility time and **(C)** number of line crossings. Novel object recognition test showing **(D)** total exploration time **(E)** exploration index; **(F)** recognition index (%) after a 1 h retention interval and **(G)** total exploration time, **(H)** exploration index; **(I)** recognition index (%) after a 24 h retention interval. Data are presented as mean ± SEM (*n* = 8). *p < 0.05, **p < 0.01 and ***p < 0.001 versus the STD group; #*p* < 0.05, ##*p* < 0.01 and ###*p* < 0.001 versus the HFD group; $*p* < 0.05 and $$$*p* < 0.001 versus HFD + OFIF group.

To evaluate the impact of OFIF on cognitive function in obese mice, behavioral assessments were performed using the open field test (OFT) and the object recognition test (ORT). In the OFT, spontaneous locomotor activity and exploratory behavior were assessed by measuring total travelled distance, immobility time, and number of line crossings. Chronic HFD administration significantly impaired locomotor activity, as evidenced by the marked reduction in total travelled distance in HFD mice compared with STD controls (Figure 3A). OFIF supplementation partially restored locomotor performance, although the effect did not reach statistical significance relative to the HFD group (Figure 3A). Notably, HFD + ATB + OFIF mice exhibited markedly reduced locomotion compared with HFD + OFIF animals (Figure 3A). Similarly, HFD mice displayed a pronounced increase in immobility time compared with STD controls, suggesting reduced spontaneous exploratory activity (Figure 3B). OFIF supplementation significantly reduced immobility time relative to HFD mice (Figure 3B). Interestingly, antibiotic treatment did not abolish this beneficial effect, as HFD + ATB + OFIF mice maintained low immobility levels (Figure 3B). Consistent with the total distance data, the number of line crossings was significantly reduced in HFD and HFD + ATB + OFIF mice compared with STD controls (Figure 3C). Although OFIF supplementation improved exploratory activity, this effect did not reach statistical significance (Figure 3C).

To evaluate short- and long-term recognition memory, mice were subjected to the ORT at 1 h and 24 h after training. HFD-fed mice showed an overall reduction in object exploratory behavior, which reached statistical significance only at the 24 h retention time (Figures 3D,G). OFIF supplementation significantly increased total exploration time compared with the HFD group at both retention intervals (Figures 3D,G). In contrast, HFD + ATB + OFIF mice displayed a marked reduction in exploration time relative to HFD + OFIF animals (Figures 3D,G). Analysis of recognition memory performance revealed a severe cognitive impairment in HFD mice, as demonstrated by the significant reduction in exploration index compared with STD controls at both retention times (Figures 3E,H), indicating impaired discrimination of the novel object. OFIF supplementation significantly improved the exploration index, restoring values toward those observed in STD mice (Figures 3E,H). Antibiotic treatment attenuated this beneficial effect, with HFD + ATB + OFIF mice showing a lower exploration index than HFD + OFIF animals, reaching statistical significance at the 24 h retention interval (Figures 3E,H). Similarly, recognition index (RI%) was significantly decreased in HFD mice compared with STD controls at the 1 h retention interval, confirming impaired recognition memory (Figure 3F). OFIF supplementation did not improve RI% at both retention intervals (Figures 3F,I). Moreover, microbiota depletion in HFD + ATB + OFIF mice further impaired memory retention compared with the HFD + OFIF group (Figures 3F,I).

### 3.4 OFIF on Alzheimer’s disease gene expression profiles

**Figure 4:**
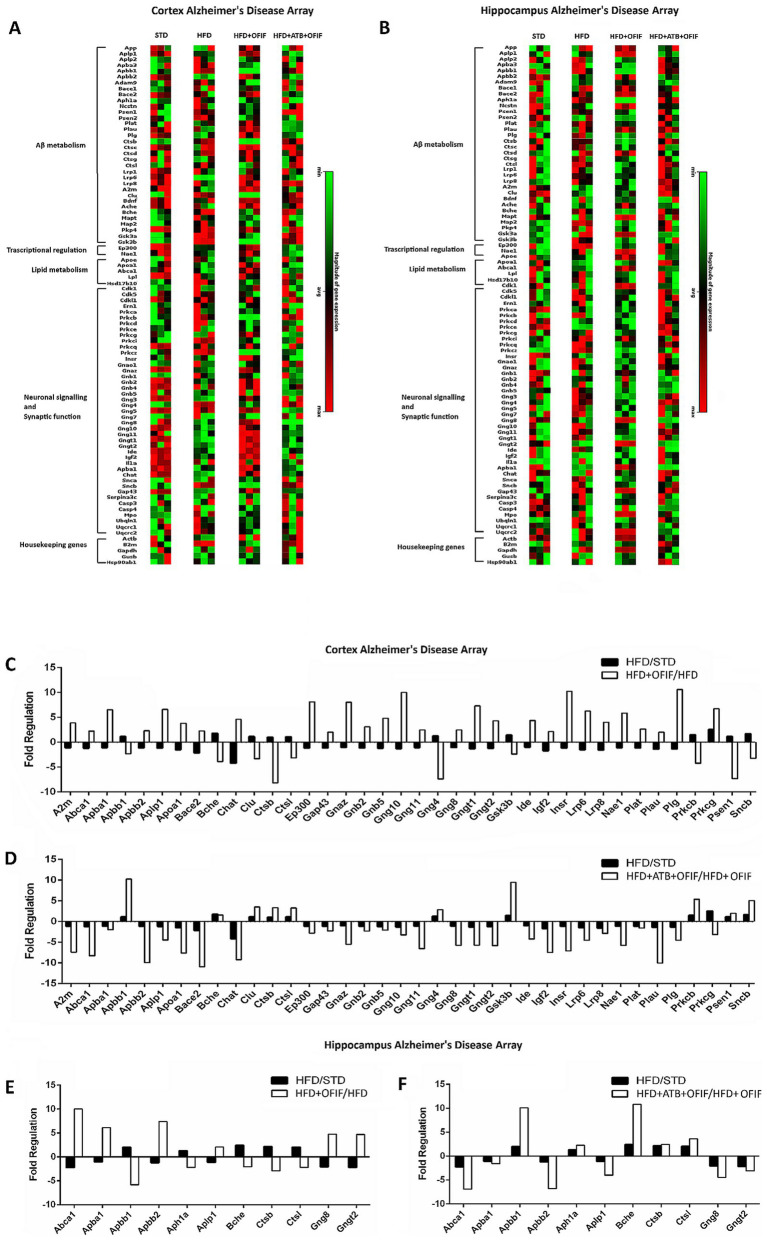
Impact of OFIF on Alzheimer’s disease gene expression profiles in different groups of mice (*n* = 3). Heat maps showing clustering of relative gene expression across all tested samples in the **(A)** cortex and **(B)** hippocampus; rows represent individual genes and columns represent individual biological samples. Bar graphs showing representative genes with at least a 2-fold change in expression in HFD + OFIF mice compared with HFD control mice in the **(C)** cortex and **(E)** hippocampus, and in HFD + OFIF mice compared with HFD + ATB + OFIF mice in the **(D)** cortex and **(F)** hippocampus. Heat map generation and fold-change analyses were performed using GeneGlobe’s online platform (RT^2^ Profiler PCR Data Analysis).

Based on the observed differences in brain morphology and cognitive functions, we performed PCR array analyses to investigate potential changes in the expression of Alzheimer’s disease-related genes in the cortex and hippocampus of the different experimental groups. In the cortex, HFD mice displayed a distinct transcriptional profile compared with STD animals, with dysregulation of several genes involved in Aβ metabolism, synaptic function, lipid metabolism, transcriptional regulation, and neuronal signaling (Figures 4A,C). Relative to HFD mice, HFD + OFIF animals showed significant modulation of 38 genes, including 28 upregulated and 10 downregulated genes. The modulated genes mainly belonged to pathways related to Aβ metabolism (*A2m*, *Apbb1*, *Apbb2*, *Bace2*, *Bche*, *Ctsb*, *Ctsl*, *Clu*, *Gsk3b*, *Plat*, *Plau*, *Plg*, *Aplp1*, *Lrp6*, *Lrp8*, *Psen1*), synaptic function (*Apba1*, *Chat*, *Sncb*, *Gap43*), lipid metabolism (*Abca1*, *Apoa1*), transcriptional regulation (*Ep300*, *Nae1*), and neuronal signaling (*Prkcb*, *Prkcg*, *Insr*, *Gnaz*, *Gnb2*, *Gnb5*, *Gng4, Gng8, Gng10*, *Gng11*, *Gngt1*, G*ngt2*, *Ide, Igf2*) (Figures 4A,C). In contrast, in HFD + ATB + OFIF mice, the expression profile of most of these genes was comparable to that observed in HFD mice, suggesting that the transcriptional effects induced by OFIF in the cortex may be largely dependent on the presence of the gut microbiota (Figures 4A,D). HFD also induced significant transcriptional alterations compared with STD mice in the hippocampus (Figures 4B,E). Relative to HFD control animals, HFD + OFIF mice showed significant changes in the expression of 11 genes. Among these, six genes were upregulated (*Abca1*, *Apba1*, *Apbb2*, *Aplp1*, *Gng8*, and *Gngt2*), while five genes were downregulated (*Apbb1*, *Aph1a*, *Bche*, *Ctsb*, and *Ctsl*) (Figures 4B,E). Notably, antibiotic treatment markedly attenuated all these transcriptional changes, with HFD + ATB + OFIF mice displaying gene expression levels comparable to those of HFD animals (Figures 4B,F). These data further support a role for the gut microbiota in mediating the hippocampal gene expression changes induced by OFIF. To validate the PCR array results, the expression of four selected genes (*Abca1*, *Apbb1*, *Apbb2*, and *Bche*) was assessed by quantitative real-time PCR in the cortex and hippocampus. Consistent with the array data, *Apbb1* and *Bche* were significantly upregulated, whereas *Abca1* and *Apbb2* were downregulated in the HFD and HFD + ATB + OFIF group compared to STD. Notably, treatment with OFIF reversed these alterations, restoring gene expression levels toward STD values (Supplementary Figure S1).

### 3.5 OFIF and gut microbiota

**Figure 5:**
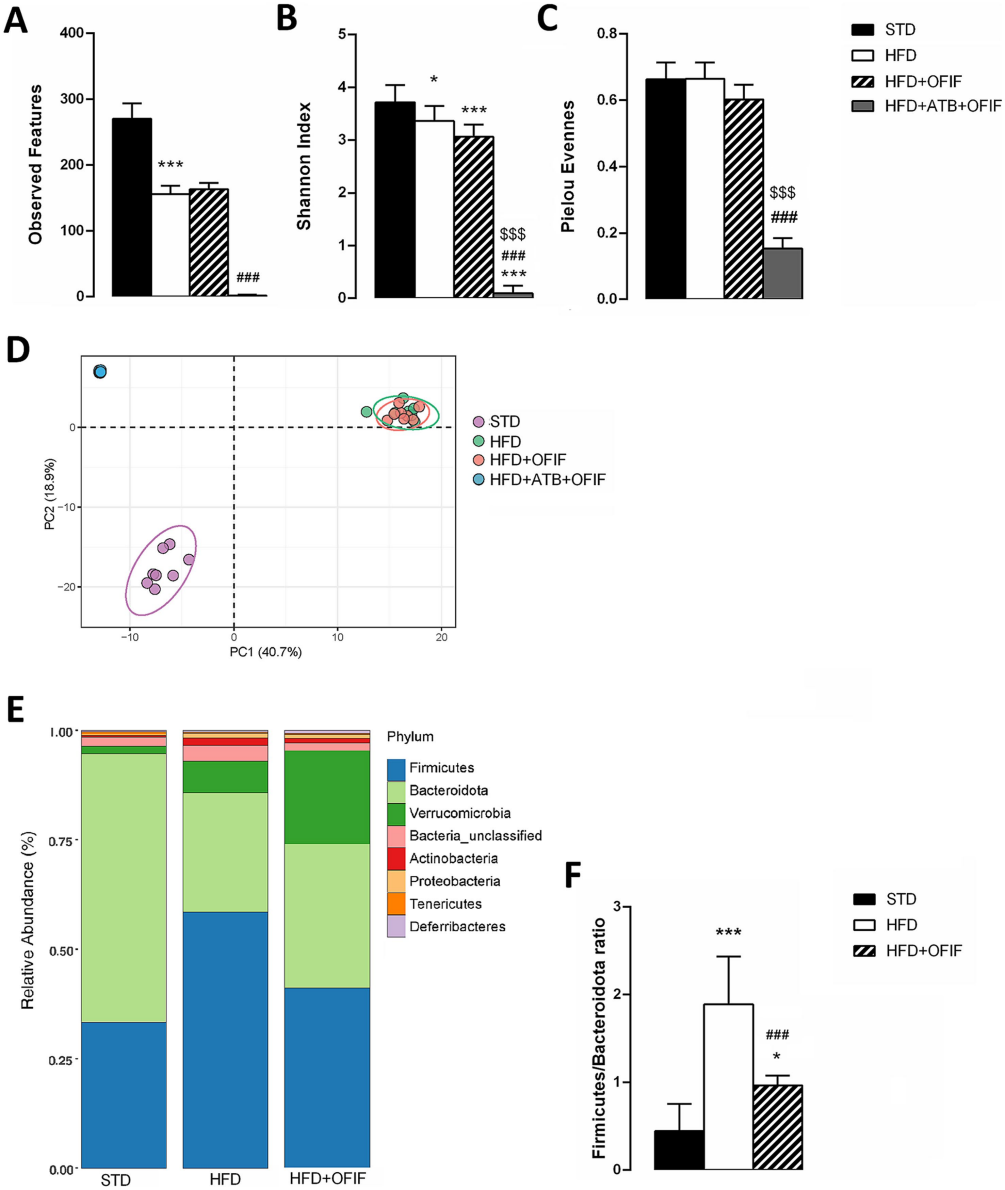
Impact of OFIF on gut microbiota composition in the different groups of mice. Alpha diversity of the gut microbiota in mice measured by **(A)** observed features, **(B)** Shannon index, and **(C)** Pielou evenness; **(D)** PCoA of each fecal sample bacterial profile; **(E)** composition of the gut microbiota at phylum level; **(F)** ratio of firmicutes to bacteroidetes. Data are presented as mean ± SEM (*n* = 8) *p < 0.05, ***p < 0.001 versus STD mice; ###*p* < 0.001 versus HFD group; $$$*p* < 0.001 versus HFD + OFIF.

Analysis of fecal bacterial communities revealed that mice fed STD exhibited significantly higher microbial *α*-diversity, as assessed by Observed features and the Shannon index, compared with the HFD group, whereas no significant differences were detected between the two groups using the Pielou evenness index (Figures 5A–C). No significant differences in α-diversity indices were observed between HFD and HFD + OFIF mice (Figures 5A–C). In contrast, as expected, treatment with broad-spectrum antibiotics profoundly altered the gut microbiota, resulting in a marked reduction of α-diversity in HFD + ATB + OFIF mice compared with all other experimental groups (Figures 5A–C).

Principal component analysis (PCoA) based on amplicon sequence variant (ASV) abundance profiles revealed distinct clustering of gut microbial communities among experimental groups. Specifically, the STD group clustered separately from all HFD-fed groups, while antibiotic treatment shifted the microbial composition away from both STD and HFD clusters (Figure 5D), indicating a substantial alteration of community structure.

At the phylum level, mice fed HFD displayed a gut microbiota dominated by *Firmicutes*, *Bacteroidota*, and *Verrucomicrobia*, with lower relative abundances of *Actinobacteria*, *Proteobacteria*, *Tenericutes*, and *Deferribacteres* (Figure 5E). Compared with the HFD group, STD fed mice exhibited lower relative abundances of *Firmicutes* and *Verrucomicrobia* and a higher abundance of *Bacteroidota*. Similarly, HFD + OFIF mice showed a significant reduction in *Firmicutes* and an increase in *Bacteroidota* and *Verrucomicrobia* relative to HFD mice (Figure 5E), resulting in a decreased *Firmicutes/Bacteroidota* ratio (Figure 5F). The relative abundances of minor phyla, including *Actinobacteria*, *Proteobacteria*, *Tenericutes*, and *Deferribacteres*, remained low across groups and did not show evident changes between STD, HFD and HFD + OFIF mice (Figure 5E).

**Figure 6:**
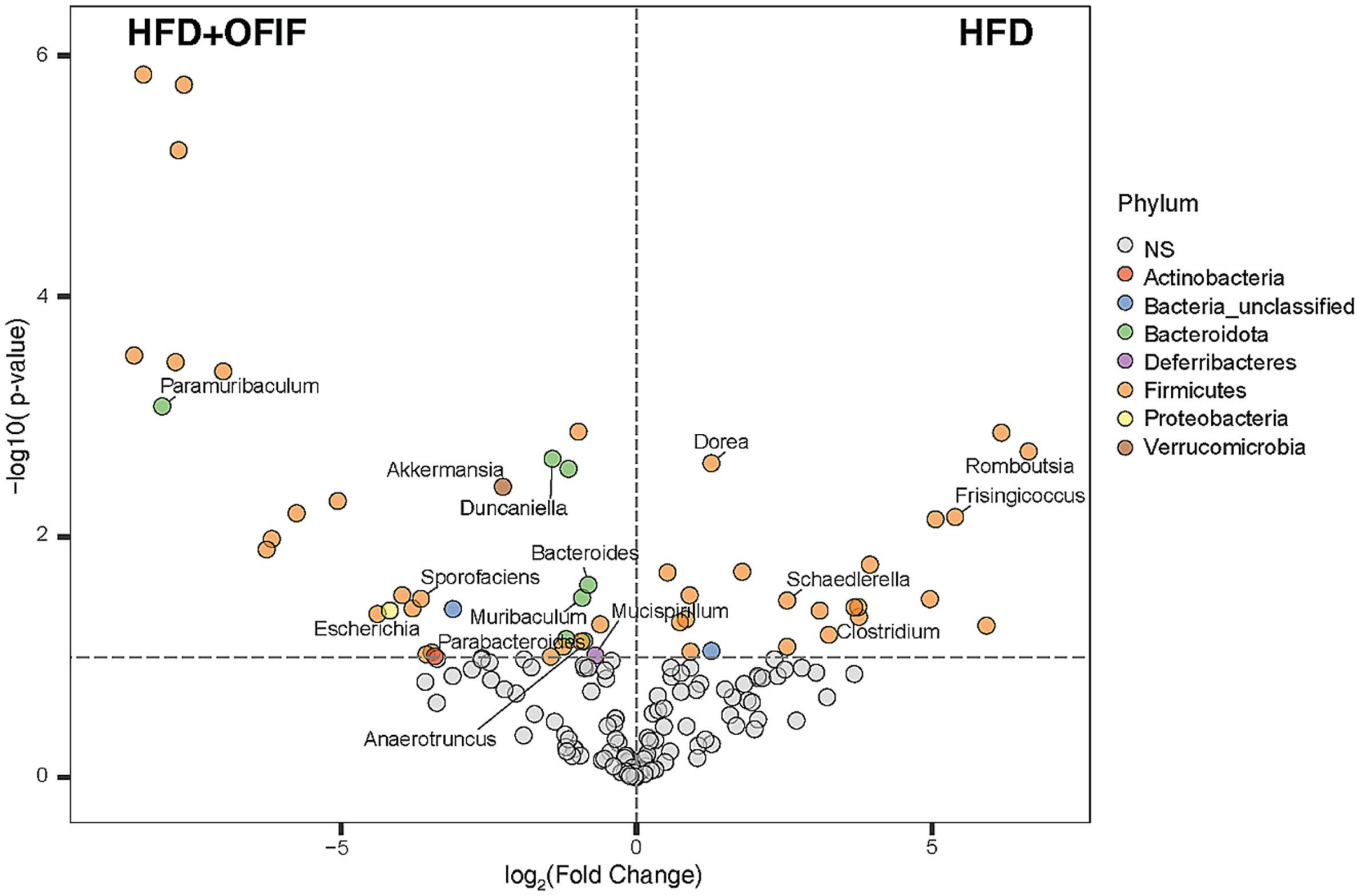
Volcano plot showing bacterial taxa identified by ANCOM-BC between the HFD + OFIF and HFD groups. The x-axis represents the log2 fold change between groups, while the y-axis represents the −log10(*p*-value). Each point corresponds to a bacterial taxon and significantly different taxa (*p* < 0.1) are highlighted and colored according to their phylum. Among these, 15 taxa could be confidently assigned at the genus level and are labelled in the figure. Significant unclassified or unidentified taxa are also displayed but are not labelled.

To further investigate changes in bacterial abundance induced by OFIF supplementation, we performed a population-level analysis of microbiota composition with bias correction (ANCOM-BC) at the genus level and visualized differential abundance between HFD and HFD + OFIF groups using a volcano plot (Figure 6).

ANCOM-BC identified several bacterial taxa with significantly different abundances between HFD and HFD + OFIF mice. Among these, 15 could be confidently assigned at the genus level and were therefore considered for genus-level interpretation (Figure 6). Genera enriched in the HFD + OFIF group, appearing on the left side of the volcano plot, included *Duncaniella, Muribaculum, Paramuribaculum, Bacteroides, Parabacteroides, Akkermansia, Anaerotruncus, Mucispirillum, Escherichia*, and *Sporofaciens*. In contrast, genera enriched in the HFD group, appearing on the right side of the volcano plot, included *Clostridium, Dorea, Frisingicoccus, Schaedlerella*, and *Romboutsia*.

To explore whether changes in gut microbiota induced by OFIF supplementation were associated with neural and molecular outcomes, Spearman correlation analyses were performed between the 15 bacterial genera differentially abundant between HFD and HFD + OFIF mice and brain weight indices as well as hippocampal and cortical gene expression.

**Figure 7:**
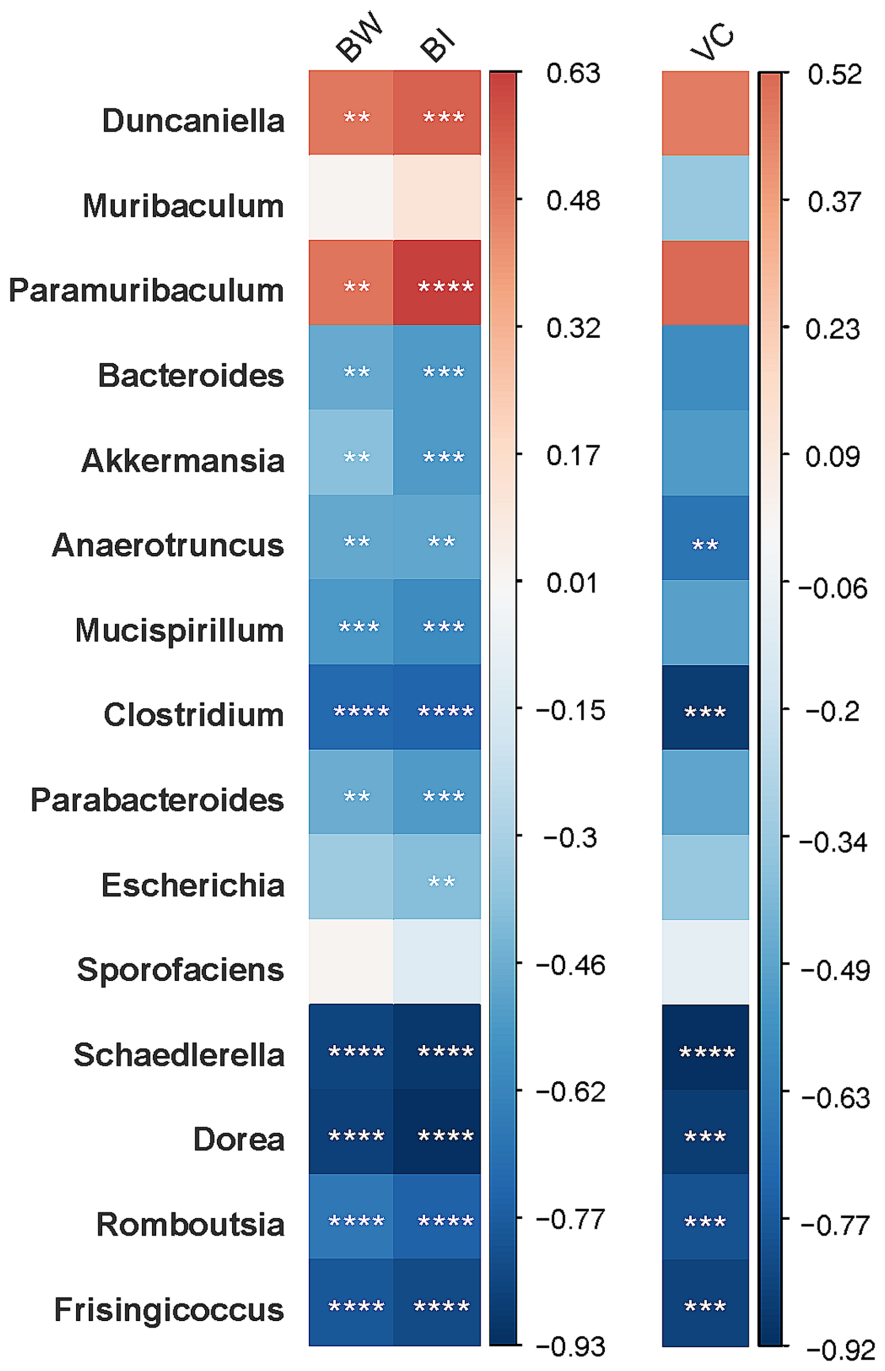
Heatmap displaying the Spearman correlation between microbiota genera significantly differing between HFD + OFIF and HFD groups and brain weight indices. The colors range from red (positive correlation) to blue (negative correlation). BW, brain weight; BI, brain index; VC, percentage of viable cells. Significant associations are shown with asterisks: ****p < 0.001, ***p < 0.01, **p < 0.05, *p < 0.1 adjusting by false discovery rate.

Genera enriched in the HFD + OFIF group generally showed positive associations with brain weight, brain index and cortical cell viability, whereas genera enriched in the HFD group were predominantly negatively correlated with these cerebral parameters (Figure 7). Among the OFIF-enriched genera, *Duncaniella* and *Paramuribaculum* exhibited the strongest positive associations with brain structural indices.

**Figure 8:**
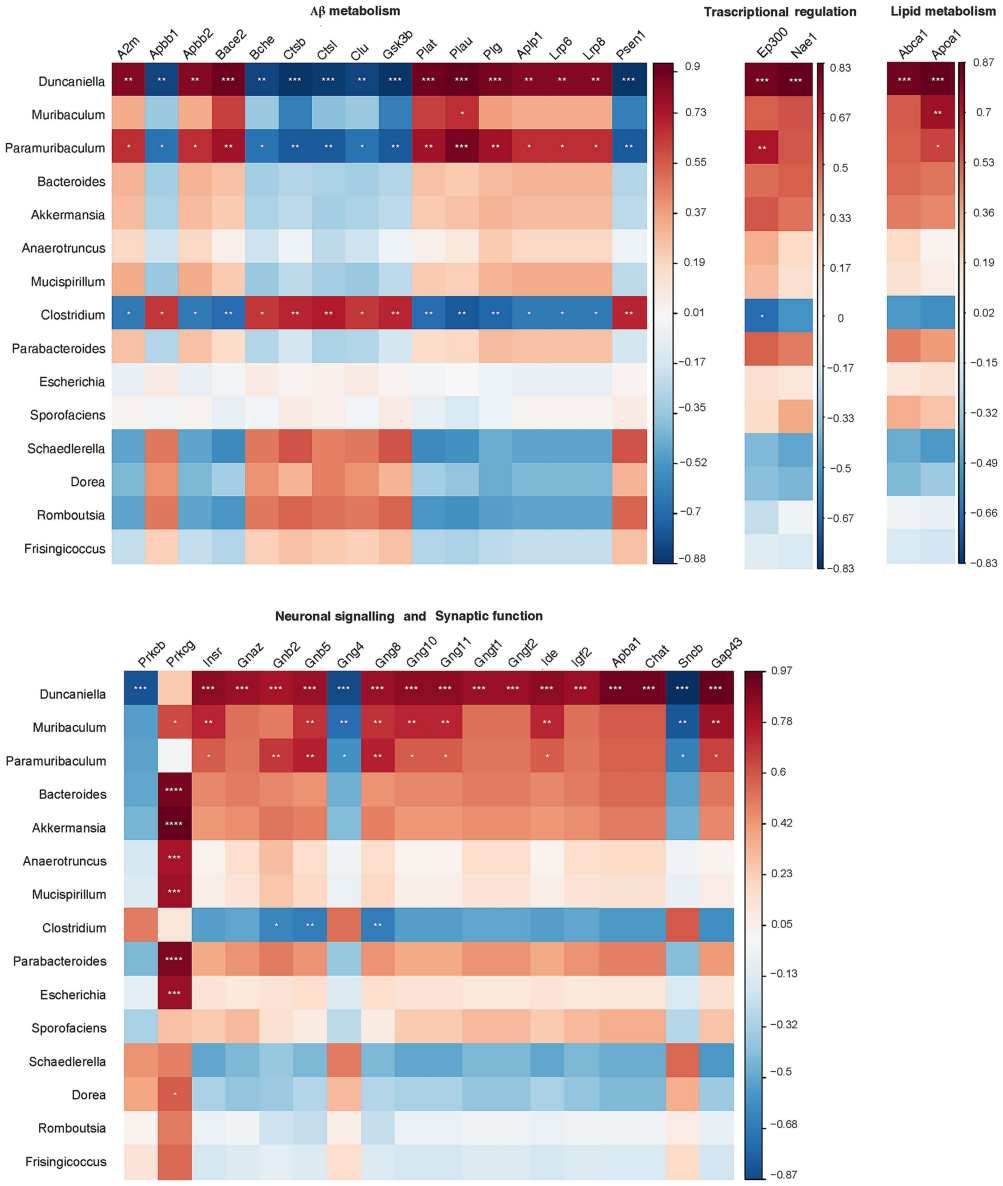
Heatmap displaying the Spearman correlation between microbiota genera significantly differing between HFD + OFIF and HFD groups and cortical gene expression. The colors range from red (positive correlation) to blue (negative correlation). Genes are grouped in five functional subclasses, Aβ metabolism, synaptic function, lipid metabolism, transcriptional regulation, and neuronal signaling. Significant associations are shown with asterisks: ****p < 0.001, ***p < 0.01, **p < 0.05, *p < 0.1 adjusting by false discovery rate.

Correlations analyses with cortical gene expression revealed distinct association patterns between bacterial genera and genes involved in Aβ metabolism, lipid metabolism, transcriptional regulation, and synaptic function/neuronal signaling (Figure 8). In particular, *Duncaniella* and *Paramuribaculum*, enriched in the HFD + OFIF group, showed widespread correlations with genes across these functional categories, whereas Clostridium, enriched in the HFD group, displayed correlations in the opposite direction, particularly with genes related to Aβ metabolism.

**Figure 9:**
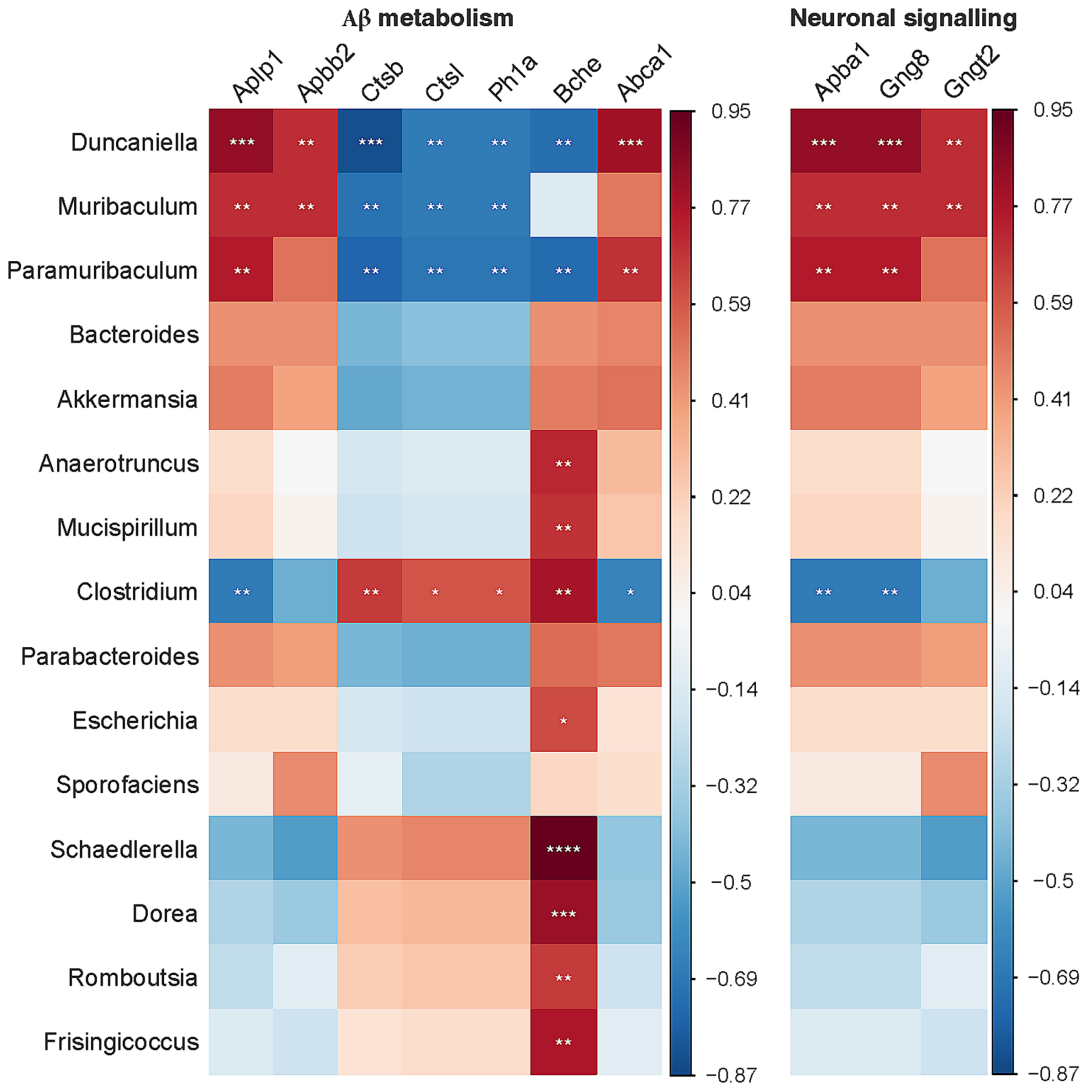
Heatmap displaying the Spearman correlation between microbiota genera significantly differing between HFD + OFIF and HFD groups and hippocampal gene expression. The colors range from red (positive correlation) to blue (negative correlation). Genes are grouped in three functional subclasses, Aβ metabolism, lipid metabolism and neuronal signaling. Significant associations are shown with asterisks: ****p < 0.001, ***p < 0.01, **p < 0.05, *p < 0.1 adjusting by false discovery rate.

Similarly, correlations with hippocampal gene expression demonstrated that OFIF-enriched genera, particularly *Duncaniella, Muribaculum*, and *Paramuribaculum*, were associated with genes involved in Aβ metabolism and neuronal signaling, while HFD-enriched genera exhibited an opposite correlation pattern. In addition, several HFD-enriched genera showed positive correlations with Bche (Figure 9).

## 4 Discussion

The present study provides evidence for sustained neuroprotective effects by supplementation with OFIF in HFD mice and for a key role of the gut microbiota in mediating these beneficial effects.

Epidemiological evidence suggests a potential correlation between metabolic syndrome, a cluster of cardiovascular risk factors including obesity, insulin resistance, dyslipidemia, and an increased risk of cognitive disorders (22–24). Moreover, studies on animal models report that HFD causes neuronal disorders such as memory alterations, mood changes, anxiety, and depression (25) and it may contribute to neurodegenerative diseases like AD and Parkinson’s disease (26, 27). Dysbiosis is present in obesity conditions, and alterations in gut microbiota composition are now considered major contributors to AD-like cognitive deficits (14).

We investigated if long-term supplementation with OFIF may have a positive impact on the brain health in HFD mice and explored the role of the gut microbiota on the OFIF-driven neuroprotection using HFD mice treated with antibiotics. We used the whole fruit to mimic a functional food intervention, rather than to attribute the observed effects to specific phytochemicals, focusing instead on the integrated impact of fruit consumption, which more plausibly modulates the gut microbiota given the complex nutritional matrix of the fruit, including fiber, sugars, and other bioactive compounds. Indeed, *Opuntia ficus-indica* fruits are a valuable source of bioactive compounds with recognized nutritional and functional properties. In particular, the yellow cultivar is characterized by the presence of organic and phenolic acids (such as citric acid, piscidic acid, ferulic acid and gluconic acid), flavonoids, the most abundant secondary metabolite class, ascorbic acid, and betalains, especially indicaxanthin, which is the predominant pigment responsible for the fruit’s yellow-orange coloration. These phytochemicals are known to contribute to the antioxidant capacity of the fruit by scavenging reactive oxygen species and reducing oxidative stress. The combined action of these bioactive compounds may explain the health-promoting effects attributed to prickly pear consumption, including antioxidant, anti-inflammatory, metabolic and gut microbiota modulatory properties (16, 28–31). In our study, as expected, HFD led to increased body weight, impaired glucose homeostasis, hyperinsulinemia, and elevated HOMA-IR. Consistent with our previous result (16), OFIF supplementation improved insulin sensitivity, as indicated by lower HOMA-IR in HFD + OFIF mice in comparison with HFD mice. Notably, HOMA-IR was decreased also in OFIF plus antibiotic-treated HFD mice. This might suggest that the improvement of insulin sensitivity is not dependent on the gut microbiota, as previously hypothesized (16). However, in HFD mice treated with cocktail of antibiotic without OFIF, HOMA-IR was 4.5 ± 0.2 *(data not shown)* supporting the hypothesis that the antibiotic treatment *per se* improve the insulin sensitivity. In according with our interpretation other studies report that antibiotic treatment may improve glucose metabolic parameters in HFD mice by reducing the production of pro-inflammatory bacterial metabolites and limiting systemic inflammation (32, 33). Therefore, the positive effect on insulin sensitivity observed in the HFD + ATB + OFIF group may derive from the direct impact of antibiotics on the gut microbiota.

Furthermore, HFD feeding resulted in reduced brain weight and brain index, accompanied by histopathological features indicative of neuronal damage, such as reduced neuronal density, cytoplasmic eosinophilia, and nuclear pyknosis. In fact, these structural brain alterations, including reduced brain mass, cortical atrophy, neuronal loss, and changes in neuronal architecture, are key hallmarks of neurodegeneration (34–36). OFIF supplementation prevented HFD-induced reduction in brain weight and preserved brain morphology and neuronal organization, suggesting a neuroprotective effect. A recent study reported that OFIF supplementation (14 mg/Kg twice a day) for 1 month mitigated cognitive alterations in HFD rats inducing beneficial effects on dysmetabolism (17). However, to our knowledge, our study is the first that provides evidence for the neuroprotective effect of OFIF in HFD-fed mice, that are dependent on the presence of intact gut-microbiota, as supported by the observation that protective effects were completely abolished following antibiotic-induced depletion of the gut microbiota. Notably, the dose used in the present study was designed to reflect a nutritionally relevant human intake, corresponding to the consumption of approximately four fruits per day (75 g lyophilized product), thereby supporting the translational relevance of the findings.

Behavioral analyses further support the hypothesis that OFIF exerts neuroprotective effects in the context of HFD-induced obesity. In agreement with previous studies, HFD-fed mice displayed reduced locomotor and exploratory activity together with impaired recognition memory, confirming that chronic exposure to an obesogenic diet negatively affects both emotional reactivity and cognitive performance, particularly hippocampal-dependent functions (37–39). OFIF supplementation partially ameliorated the behavioral deficits induced by HFD, reducing immobility time in the open field test and enhancing performance in the object recognition task. Interestingly, the beneficial effects of OFIF were more evident for exploratory and motivational behaviors than for recognition index performance, suggesting that different behavioral domains may display distinct sensitivities to nutritional and microbiota-dependent modulation. Moreover, the lack of full recovery in RI% indicates that some aspects of memory discrimination may be less responsive to the nutritional intervention or may require longer treatment duration. Importantly, depletion of gut microbiota by antibiotic administration markedly attenuated most of the beneficial effects induced by OFIF, particularly in locomotor and cognitive parameters. These findings suggest that an intact gut microbiota may contribute to the neurobehavioral effects of OFIF. Nevertheless, the present results do not identify the molecular or cellular mechanisms underlying this association, which warrant further investigation.

Neurodegeneration induced by metabolic alterations such as HFD shares several pathological features and molecular mechanisms with AD (40). Consequently, in light of the observed variations in brain structure and cognitive function, PCR array analyses were carried out to examine possible changes in the expression of genes involved in Alzheimer Disease-related molecular pathways in the cortex and hippocampus of the different experimental groups. At the molecular level, both in cortex and hippocampus, OFIF modulated the expression of multiple Alzheimer’s disease (AD)-related genes, suggesting a potential role of the fruit in modulating brain lipid metabolism, APP processing and synaptic functionality-mechanisms closely implicated in the pathogenesis of neurodegeneration and AD (40).

Specifically, our findings suggest that OFIF induces a broad transcriptional reprogramming in the cortex of HFD mice, affecting multiple genes that are critically involved in neurodegeneration, synaptic function, and metabolic regulation. Notably, the modulation of genes associated with Aβ metabolism (e.g., *Bace2, Psen1, Ctsb, Ctsl, Lrp8*) suggests that OFIF may influence amyloidogenic processing and clearance pathways. Alterations in these genes have been directly linked to amyloid deposition and cognitive decline in experimental models of AD (41–43). In parallel, the upregulation of genes involved in synaptic function and neuronal plasticity (e.g., *Apba1, Chat, Gap43*) suggests a potential enhancement of synaptic integrity and neurotransmission. The observed regulation of genes related to lipid metabolism (*Abca1, Apoa1*) and insulin signaling (*Insr, Igf2*) also is indicative of improved metabolic homeostasis within the cortex. This is particularly relevant in the context of HFD-induced neurodegeneration, where metabolic dysfunction represents a key driver of cognitive impairment (44). The transcriptional profile observed in the hippocampus further reinforces the hypothesis that OFIF exerts a neuroprotective action. Although the number of differentially expressed genes was more limited compared to the cortex, the affected targets converge on AD-related molecular pathways and synaptic function. In particular, the upregulation of *Abca1* supports a potential enhancement of cholesterol efflux and lipid homeostasis in the hippocampus, a mechanism known to facilitate A*β* clearance and reduce amyloid burden (45). Similarly, the increased expression of *Abca1*, *Apba1, Apbb2,* and *Aplp1*, all involved in APP trafficking and processing suggests a shift toward non-amyloidogenic or regulatory pathways that may limit toxic A*β* production. On the other hand, the downregulation of *Apbb1* and *Aph1a*, key components of the amyloidogenic processing machinery, suggests a reduction in Aβ generation, reflecting a protective shift away from pathogenic APP cleavage. Concurrently, the upregulation of *Gng8* and *Gngt2*, components of G protein–coupled receptor signaling, suggests an enhancement of G protein-coupled receptor signaling, which is essential for synaptic transmission, plasticity, and memory formation. The reduced expression of *Bche* could suggest a potential improvement in cholinergic neurotransmission, which is typically impaired in AD. This aligns with hypothesis that modulation of cholinergic pathways contributes to cognitive resilience (46). Finally, the downregulation of lysosomal cathepsins (*Ctsb* and *Ctsl*) suggests attenuation of neuroinflammatory and apoptotic processes, as these proteases are recognized as mediators of both aberrant APP processing and neurodegeneration (42).

Interestingly, these transcriptional changes were largely abolished in antibiotic-treated mice, suggesting that gut microbiota is required for OFIF-driven modulation of AD-related molecular pathways. Therefore, this finding supports a microbiota-dependent contribution to the neuroprotective effects of OFIF.

Gut microbiota analysis in fecal samples of the different animal groups was performed to assess the composition. Microbiota profiling revealed HFD-induced dysbiosis. Consistently with previous reports (31, 47, 48), 16S rRNA sequencing revealed reduced microbial diversity and altered taxonomic composition, as indicated by changes in *α*- and β-diversity indices and increased Firmicutes/Bacteroidota ratio. Specifically, HFD decreased Bacteroides, bacteria mediating generally host beneficial functions, and it increased Firmicutes, bacteria with detrimental functions. In line with these results, other studies in animal models and humans have shown an increase in the ratio of Firmicutes to Bacteroides after HFD feeding (48). Moreover, HFD mice showed increased abundance of the bacterial genera, such as *Clostridium*, *Dorea* and *Romboutsia*, frequently linked to obese-like features, metabolic disorders and systemic inflammation, conditions that contribute to cognitive impairment (49, 50). OFIF supplementation partially prevented HFD-induced dysbiosis. In fact, although α-diversity and β-diversity were not restored, OFIF induced compositional shifts, notably reducing significantly the Firmicutes/Bacteroidota ratio and enriching genera such as *Duncaniella*, *Muribaculum*, *Paramuribaculum*, *Bacteroides*, *Parabacteroides*, and *Akkermansia*. Among these taxa, *Akkermansia* and *Parabacteroides* have been reported to reduce neuroinflammation and improve cognitive function (51, 52). Moreover, the abundance of *Muribaculum* has been associated with modulation of the gut–brain axis through effects on host neurotransmitter synthesis, immune responses, and intestinal barrier function mediated by microbial metabolites such as short-chain fatty acids (SCFAs) (53). Similarly, *Paramuribaculum* and *Duncaniella* participate in the fermentation of complex carbohydrates, producing SCFAs and contributing to immune regulation and the reduction of inflammation (54).

Correlation analyses further supported the functional relevance of the gut microbiota modulation. OFIF-enriched genera, particularly *Duncaniella* and *Paramuribaculum*, were positively associated with brain weight, and beneficial gene expression profiles, whereas HFD-associated genera showed opposite correlations. Notably, *Duncaniella* exhibited significant associations across multiple cortical gene pathways, suggesting a central role in mediating gut–brain communication leading to improvement of Aβ metabolism, synaptic functionality, and lipid metabolism. On the other hand, emerging evidence suggests that *Duncaniella,* a bacterial genus within the family Muribaculaceae, may play an important role in carbohydrate metabolism, production of SCFAs, and host metabolic homeostasis, with downstream effects on immune and neurological function (55).

We are conscious that this study has some limitations. Including an OFIF-supplemented STD group would have provided additional information on the effects of OFIF under metabolically unaltered conditions. However, the study design was constrained by the need to comply with the 3Rs principle (Replacement, Reduction, and Refinement), with particular emphasis on the Reduction principle aimed at minimizing the number of animals used. While strong associations between microbiota composition and neurobiological outcomes were identified, the specific microbial metabolites and signaling pathways mediating these effects were not directly assessed. In addition, the use of broad-spectrum antibiotics, while effective in demonstrating microbiota dependence, does not allow identification of specific bacterial species responsible for the observed benefits. Another limitation is represented by the exclusive use of male mice, that we chose to reduce biological variables and potential hormonal fluctuations, but it does not allow generalizability of obtained results.

In conclusion, our findings demonstrate that the neuroprotective effects of long-term OFIF supplementation are dependent on the presence of an intact gut microbiota, although, the molecular mediators underlying this gut-brain interaction remain to be elucidated. Collectively, these findings suggest that increased consumption of *Opuntia ficus-indica* fruit may represent a promising prebiotic strategy to mitigate obesity-associated cognitive decline.

## Data Availability

The original contributions presented in the study are publicly available. These data can be found in the NCBI Sequence Read Archive (SRA) under Study accession number SRP720171 and BioProject accession number PRJNA1498323.

## Footnotes

1
https://geneglobe.qiage.com/ca/analyze

## Glossary

AD: Alzheimer’s disease
ANCOM-BC: Analysis of Compositions of Microbiomes with Bias Correction
ASV: Amplicon Sequence Variant
ATB: Antibiotic cocktail
cDNA: Complementary DNA
ELISA: Enzyme-Linked Immunosorbent Assay
H&E: Hematoxylin and Eosin
HFD: High-Fat Diet
HOMA-IR: Homeostatic Model Assessment of Insulin Resistance
OFIF: *Opuntia ficus-indica* Fruit
OFT: Open Field Test
ORT: Object Recognition Test
PBS: Phosphate-Buffered Saline
PCoA: Principal Coordinates Analysis
PCR: Polymerase Chain Reaction
qPCR: Quantitative Real-Time Polymerase Chain Reaction
RNA: Ribonucleic Acid
RT^2^ PCR Array: Reverse Transcription Profiler PCR Array
SEM: Standard Error of the Mean
STD: Standard Diet

## References

[1] Pugazhenthi S Qin L Reddy PH. Common neurodegenerative pathways in obesity, diabetes, and Alzheimer's disease. Biochim Biophys Acta Mol basis Dis. (2017) 1863:1037–45. doi: 10.1016/j.bbadis.2016.12.014, 27156888 PMC5344771

[2] Ruan Y Tang J Guo X Li K Li D. Dietary fat intake and risk of Alzheimer’s disease and dementia: a meta-analysis of cohort studies. Curr Alzheimer Res. (2018) 15:869–76. doi: 10.2174/1567205015666180613112237 29701155

[3] Valentin-Escalera J Leclerc M Calon F. High-fat diets in animal models of Alzheimer's disease: how can eating too much fat increase Alzheimer's disease risk? J Alzheimer's Dis. (2024) 97:977–1005. doi: 10.3233/JAD-230, 38217592 PMC10836579

[4] Scheltens P De Strooper B Kivipelto M Holstege H Chételat G Teunissen CE. Alzheimer’s disease. Lancet. (2021) 397:1577–90. doi: 10.1016/S0140-6736(20)32205-4 33667416 PMC8354300

[5] De-Paula VJ Radanovic M Diniz BS Forlenza OV. Alzheimer’s disease. Subcell Biochem. (2012) 65:329–52. doi: 10.1007/978-94-007-5416-4_14 23225010

[6] Kamatham PT Shukla R Khatri DK Vora LK. Pathogenesis, diagnostics, and therapeutics for Alzheimer’s disease: breaking the memory barrier. Ageing Res Rev. (2024) 101:102481. doi: 10.1016/j.arr.2024.102481, 39236855

[7] Wong Zhang DE Tran V Vinh A Dinh QN Drummond GR Sobey CG . Pathophysiological links between obesity and dementia. NeuroMolecular Med. (2023) 25:451–6. doi: 10.1007/s12017-023-08746-1, 37086380 PMC10721659

[8] Kim DG Krenz A Toussaint LE Maurer KJ Robinson SA Yan A . Non-alcoholic fatty liver disease induces signs of Alzheimer’s disease (AD) in wildtype mice and accelerates pathological signs of AD in an AD model. J Neuroinflammation. (2016) 13:1. doi: 10.1186/s12974-015-0467-5, 26728181 PMC4700622

[9] Biyong EF Alfos S Dumetz F Helbling JC Aubert A Brossaud J . Dietary vitamin a supplementation prevents early obesogenic diet-induced microbiota, neuronal and cognitive alterations. Int J Obes. (2021) 45:588–98. doi: 10.1038/s41366-020-00723-z, 33223517

[10] Galizzi G Deidda I Amato A Calvi P Terzo S Caruana L . Aphanizomenon flos-aquae extract prevents neurodegeneration in the HFD mouse model by modulating astrocytes and microglia activation. Int J Mol Sci. (2023) 24:4731. doi: 10.3390/ijms24054731 36902167 PMC10003388

[11] Terzo S Calvi P Nuzzo D Picone P Allegra M Mulè F . Long-term ingestion of Sicilian black bee chestnut honey and/or D-limonene counteracts brain damage induced by high fat-diet in obese mice. Int J Mol Sci. (2023) 24:3467. doi: 10.3390/ijms24043467, 36834882 PMC9966634

[12] Massaro A Calvi P Restivo I Giardina M Mulè F Tesoriere L . Kumquat fruit administration counteracts Dysmetabolism-related neurodegeneration and the associated brain insulin resistance in the high-fat diet-fed mice. Int J Mol Sci. (2025) 26:3077. doi: 10.3390/ijms26073077, 40243721 PMC11988715

[13] Terzo S Amato A Mulè F. From obesity to Alzheimer’s disease through insulin resistance. J Diabetes Complicat. (2021) 35:108026. doi: 10.1016/j.jdiacomp.2021.108026, 34454830

[14] Deng M Tang F Zhu Z. Altered cognitive function in obese patients: relationship to gut flora. Mol Cell Biochem. (2025) 480:3553–67. doi: 10.1007/s11010-024-05201-y, 39937394 PMC12095350

[15] Wang Y Wang K Yan J Zhou Q Wang X. Recent Progress in research on mechanisms of action of natural products against Alzheimer's disease: dietary plant polyphenols. Int J Mol Sci. (2022) 23:13886. doi: 10.3390/ijms232213886, 36430365 PMC9695301

[16] Terzo S Calvi P Restivo I Massaro A Tesoriere L Fernández-Real JM . *Opuntia ficus-indica* fruit consumption improves insulin resistance in mice with diet-induced obesity. J Sci Food Agric. (2025) 105:7868–80. doi: 10.1002/jsfa.70038, 40714989 PMC12509048

[17] Di Majo D Ricciardi N Di Liberto V Allegra M Frinchi M Urone G . The remarkable impact of *Opuntia ficus-indica* fruit administration on metabolic syndrome: Correlations between cognitive functions, oxidative stress and lipid dysmetabolism in the high-fat, diet-fed rat model. Biomed Pharmacother. (2024) 177:117028. doi: 10.1016/j.biopha.2024.117028 38959603

[18] Gambino G Frinchi M Giglia G Scordino M Urone G Ferraro G . Impact of “Golden” tomato juice on cognitive alterations in metabolic syndrome: insights into behavioural and biochemical changes in a high-fat diet rat model. J Funct Foods. (2024) 12:105964. doi: 10.1016/j.jff.2023.105964

[19] Takase K Tsuneoka Y Oda S Kuroda M Funato H. High-fat diet feeding alters olfactory-, social-, and reward-related behaviors of mice independent of obesity. Obesity. (2016) 24:886–94. doi: 10.1002/oby.21441, 26890672

[20] Inayat M Cruz-Sanchez A Thorpe HHA Frie JA Richards BA Khokhar JY . Promoting and optimizing the use of 3D-printed objects in spontaneous recognition memory tasks in rodents: a method for improving rigor and reproducibility. eNeuro. (2021) 8:ENEURO.0319-21.2021. doi: 10.1523/ENEURO.0319-21.2021, 34503967 PMC8489023

[21] Takahashi S Tomita J Nishioka K Hisada T Nishijima M . Development of a prokaryotic universal primer for simultaneous analysis of bacteria and archaea using next-generation sequencing. PLoS One. (2014) 9:e105592. doi: 10.1371/journal.pone.0105592 25144201 PMC4140814

[22] Wang X Ji L Tang Z Ding G Chen X Lv J . The association of metabolic syndrome and cognitive impairment in Jidong of China: a cross-sectional study. BMC Endocr Disord. (2021) 21:40. doi: 10.1186/s12902-021-00705-w, 33663435 PMC7934472

[23] Ji X Zou W Fan L Zhou Z Zhu X Li X. Insulin resistance-related features are associated with cognitive decline: a cross-sectional study in adult patients with type 1 diabetes. Diabetol Metab Syndr. (2024) 16:13. doi: 10.1186/s13098-023-01249-w, 38212850 PMC10782534

[24] Chapple B Bayliss E Woodfin S Smith M Winter J Moore W. Type 3 diabetes: linking insulin resistance to cognitive decline. Diseases. (2025) 13:359. doi: 10.3390/diseases13110359, 41294899 PMC12651046

[25] Fadó R Molins A Rojas R Casals N. Feeding the brain: effect of nutrients on cognition, synaptic function, and AMPA receptors. Nutrients. (2022) 14:4137. doi: 10.3390/nu14194137, 36235789 PMC9572450

[26] Boulos C Yaghi N El Hayeck R Heraoui GN Fakhoury-Sayegh N. Nutritional risk factors, microbiota and Parkinson's disease: what is the current evidence? Nutrients. (2019) 11:1896. doi: 10.3390/nu11081896 31416163 PMC6722832

[27] Sánchez-Alegría K Arias C. Functional consequences of brain exposure to saturated fatty acids. Endocrinol Diabetes Metab. (2023) 6:e386. doi: 10.1002/edm2.386 36321333 PMC9836261

[28] Terzo S Attanzio A Calvi P Mulè F Tesoriere L Allegra M . Indicaxanthin from *Opuntia ficus-indica* fruit ameliorates glucose dysmetabolism and counteracts insulin resistance in high-fat-diet-fed mice. Antioxidants. (2021) 11:80. doi: 10.3390/antiox11010080, 35052584 PMC8773302

[29] Giraldo-Silva L Ferreira B Rosa E Dias ACP. *Opuntia ficus-indica* fruit: a systematic review of its phytochemicals and pharmacological activities. Plants. (2024) 12:543. doi: 10.3390/plants12030543 PMC9919935 36771630

[30] Gómez-García I Besné-Eseverri I Portillo MP Fernández-Quintela A Díaz LE Riezu-Boj JI . Oral administration of an *Opuntia ficus-indica* fruit extract induces changes in gut microbiota composition: relationship with its anti-obesity and anti-Steatotic effects in rats fed a high-fat high-fructose diet. Foods. (2025) 14:2891. doi: 10.3390/foods14162891, 40870803 PMC12385880

[31] Terzo S Amato A Calvi P Giardina M Nuzzo D Picone P . Positive impact of indicaxanthin from *Opuntia ficus-indica* fruit on high-fat diet-induced neuronal damage and gut microbiota dysbiosis. Neural Regen Res. (2026) 21:324–32. doi: 10.4103/NRR.NRR-D-23-02039, 39314163 PMC12094550

[32] Carvalho BM Guadagnini D Tsukumo DML Schenka AA Latuf-Filho P Vassallo J . Modulation of gut microbiota by antibiotics improves insulin signalling in high-fat fed mice. Diabetologia. (2012) 55:2823–34. doi: 10.1007/s00125-012-2648-4, 22828956

[33] Kumar S Raj VS Ahmad A Saini V. Amoxicillin modulates gut microbiota to improve short-term high-fat diet induced pathophysiology in mice. Gut Pathog. (2022) 14:40. doi: 10.1186/s13099-022-00513-0, 36229889 PMC9563906

[34] Gonzales MM Insel PS Nelson C Tosun D Mattsson N Mueller SG . Cortical atrophy is associated with accelerated cognitive decline in mild cognitive impairment with subsyndromal depression. Am J Geriatr Psychiatry. (2017) 25:980–91. doi: 10.1016/j.jagp.2017.04.011 28629965 PMC10079284

[35] Jack CRJ Bennett DA Blennow K Carrillo MC Dunn B Haeberlein SB . NIA-AA research framework: toward a biological definition of Alzheimer’s disease. Alzheimers Dement. (2018) 14:535–62. doi: 10.1016/j.jalz.2018.02.018 29653606 PMC5958625

[36] DeTure MA Dickson DW. The neuropathological diagnosis of Alzheimer’s disease. Mol Neurodegener. (2019) 14:32. doi: 10.1186/s13024-019-0333-5, 31375134 PMC6679484

[37] Jiang M Kang L Wang YL Zhou B Li HY Yan Q . Mechanisms of microbiota–gut–brain axis communication in anxiety disorders. Front Neurosci. (2024) 18:1501134. doi: 10.3389/fnins.2024.1501134, 39717701 PMC11663871

[38] Xiang Q Yu M Cai Q Hu M Rao B Liang X . Multi-omics insights into the microbiota–gut–brain axis and cognitive improvement. J Transl Med. (2024) 22:945. doi: 10.1186/s12967-024-0945 39420319 PMC11484437

[39] Ling Q Zhang J Zhong L Li X Sun T Xiang H . The role of gut microbiota in chronic restraint stress-induced cognitive deficits in mice. BMC Microbiol. (2024) 24:289. doi: 10.1186/s12866-024-0289 39095715 PMC11295512

[40] Zhang J Sima J. Lipid metabolism in Alzheimer’s disease. Aging Res. (2024) 2:9340037. doi: 10.26599/AGR.2025.9340037

[41] Huentelman M De Both M Jepsen W Piras IS Talboom JS Willeman M. Common BACE2 polymorphisms are associated with altered risk for Alzheimer's disease and CSF amyloid biomarkers in APOE ε4 non-carriers. Sci Rep. (2019) 9:9640. doi: 10.1038/s41598-019-45896-4 31270419 PMC6610620

[42] Hook G Kindy M Hook V. Cathepsin B deficiency improves memory deficits and reduces amyloid-β in hAβPP mouse models representing the major sporadic Alzheimer's disease condition. J Alzheimer's Dis. (2023) 93:33–46. doi: 10.3233/JAD-221005, 36970896 PMC10185432

[43] Bagaria J Bagyinszky E An SSA. Genetics, functions, and clinical impact of presenilin-1 (PSEN1) gene. Int J Mol Sci. (2022) 23:10970. doi: 10.3390/ijms231810970, 36142879 PMC9504248

[44] Liu P Wang ZH Kang SS Liu X Xia Y Chan CB . High-fat diet-induced diabetes couples to Alzheimer’s disease through inflammation-activated C/EBPβ/AEP pathway. Mol Psychiatry. (2022) 27:3396–409. doi: 10.1038/s41380-022-01600-z, 35546632 PMC10032575

[45] Fitz NF Cronican AA Saleem M Fauq AH Chapman R Lefterov I . Abca1 deficiency affects Alzheimer's disease-like phenotype in human ApoE4 but not in ApoE3-targeted replacement mice. J Neurosci. (2012) 32:13125–36. doi: 10.1523/JNEUROSCI.1937-12.2012, 22993429 PMC3646580

[46] Sharma K. Cholinesterase inhibitors as Alzheimer's therapeutics. Mol Med Rep. (2019) 20:1479–87. doi: 10.3892/mmr.2019.10374, 31257471 PMC6625431

[47] Terzo S Mulè F Caldara GF Baldassano S Puleio R Vitale M . Pistachio consumption alleviates inflammation and improves gut microbiota composition in mice fed a high-fat diet. Int J Mol Sci. (2020) 21:365. doi: 10.3390/ijms21010365, 31935892 PMC6981517

[48] Magne F Gotteland M Gauthier L Zazueta A Pesoa S Navarrete P . The Firmicutes/Bacteroidetes ratio: a relevant marker of gut Dysbiosis in obese patients? Nutrients. (2020) 12:1474. doi: 10.3390/nu12051474, 32438689 PMC7285218

[49] Companys J Gosalbes MJ Pla-Pagà L Calderón-Pérez L Llauradó E Pedret A . Gut microbiota profile and its association with clinical variables and dietary intake in overweight/obese and lean subjects: a cross-sectional study. Nutrients. (2021) 13:2032. doi: 10.3390/nu13062032, 34199239 PMC8231825

[50] Tian Y Yu M Bai J Chen Y Cong X Gao X. Role of oral-gut microbiota dysbiosis in regulating systemic impairment during age-related obesity: an animal study. Front Cell Infect Microbiol. (2026) 16:1781222. doi: 10.3389/fcimb.2026.1781222, 41834998 PMC12979499

[51] Wang B Pan M Yang L Xu J Ye C Li Y . *Akkermansia muciniphila* reduces neuroinflammation and aβ deposition via tryptophan metabolism in the APP/PS1 mouse model of Alzheimer's disease. Alzheimer's Res Ther. (2026) 18:41. doi: 10.1186/s13195-025-01880-x, 41715194 PMC12922204

[52] Xie J Sun Y Li Q Nie X Zhang W Dong L . Protective effects of *Parabacteroides distasonis* against high-fat diet-induced brain injury in mice. NPJ Sci Food. (2025) 9:226. doi: 10.1038/s41538-025-00226 41249170 PMC12623430

[53] Wang R He Z Li Z Pan Y Bai S. Muribaculum intestinale in brain-gut axis regulation: promises and limitations for therapeutic applications. Front Microbiol. (2026) 17:1723051. doi: 10.3389/fmicb.2026.1723051, 41800404 PMC12963064

[54] Nagano T Higashimura Y Nakano M Nishiuchi T Lelo AP. High-viscosity dietary fibers modulate gut microbiota and liver metabolism to prevent obesity in high-fat diet-fed mice. Int J Biol Macromol. (2025) 298:139962. doi: 10.1016/j.ijbiomac.2025.139962, 39826739

[55] Lamichhane G Liu J Lee SJ Lee DY Zhang G Kim Y. Curcumin mitigates the high-fat high-sugar diet-induced impairment of spatial memory, hepatic metabolism, and the alteration of the gut microbiome in Alzheimer's disease-induced (3xTg-AD) mice. Nutrients. (2024) 16:240. doi: 10.3390/nu16020240, 38257133 PMC10818691

