# Supplementary material for: Neuroprotective effects of Opuntia ficus-indica fruit in high-fat-diet-induced obese mice: evidence for a role of the gut microbiota
Source: Front Nutr. 2026 Aug 5;13:1909657. doi: 10.3389/fnut.2026.1909657 (PMC13485921; doi:10.3389/fnut.2026.1909657)
Supplement: Supplementary file 1 [file Table_1.docx]

Supplementary Material

## Supplementary Figure

**Supplementary Figure 1.** Validation of RT² Profiler PCR Array results by real-time PCR.
Relative mRNA expression levels of *Abca1, Apbb1, Apbb2,* and *Bche* in the (A) cortex and (B) hippocampus in the different group of mice. Data are presented as mean ± SEM (n = 8). *p < 0.05, **p < 0.01 and ***p < 0.001 *versus* the STD group; ###p < 0.001 *versus* the HFD group; $$ p <0.01 and $$$p < 0.001 *versus* HFD+OFIF group.
